# Current status of the lateral flow immunoassay for the detection of SARS-CoV-2 in nasopharyngeal swabs

**DOI:** 10.11613/BM.2021.020601

**Published:** 2021-06-15

**Authors:** Anita Somborac Bačura, Marija Dorotić, Leonarda Grošić, Monika Džimbeg, Slavica Dodig

**Affiliations:** Department of Medical Biochemistry and Hematology, Faculty of Pharmacy and Biochemistry, University of Zagreb, Zagreb, Croatia

**Keywords:** COVID-19, immunoassay, nasopharynx, SARS-CoV-2 antigen testing

## Abstract

Early detection of severe acute respiratory syndrome coronavirus 2 (SARS-CoV-2) and diagnosis of coronavirus disease 2019 (COVID-19) are priorities during the pandemic. Symptomatic and suspected asymptomatic individuals should be tested for COVID-19 to confirm infection and to be excluded from social interactions. As molecular testing capacity is overloaded during the pandemic, rapid antigen tests, such as lateral flow immunoassays (LFIAs), can be a useful tool as they allow greater test availability and obtain results in a very short time. This short review aims to present the analytical properties of LFIAs in the detection of SARS-CoV-2 in nasopharyngeal swabs. Lateral flow immunoassay is a method that combines thin-layer chromatography and indirect immunochemical sandwich method and allows the detection of a specific SARS-CoV-2 antigen in nasopharyngeal swabs. Swab specimens should be adequately collected and tested as soon as possible. Users should pay attention to quality control and possible interferences. Antigen tests for SARS-CoV-2 show high sensitivity and specificity in cases with high viral loads, and should be used up to five days after the onset of the first symptoms of COVID-19. False positive results may be obtained when screening large populations with a low prevalence of COVID-19 infection, while false negative results may happen due to improper specimen collection or insufficient amount of antigen in the specimen. So as to achieve reliable results, a diagnostic accuracy study of a specific rapid antigen test should be performed.

## Introduction

Since the outbreak of the coronavirus disease 2019 (COVID-19) pandemic, clinicians and laboratory scientists have been struggling to reveal the most appropriate methods for early diagnosis of the disease. COVID-19 is caused by severe acute respiratory syndrome coronavirus 2 (SARS-CoV-2), a ribonucleic acid (RNA) virus that first appeared in Wuhan, China, in late 2019 (*1*, *2*). SARS-CoV-2 mainly affects the respiratory tract, which can present as mild symptoms or progress into severe complications that can be fatal. For example, it may manifest as acute pulmonary infection, coagulation disorders or gastrointestinal symptoms (most commonly diarrhea and nausea) (*3*-*5*). SARS-CoV-2 spreads easily among people who are in close contact. Close contact is defined as being within 1.83 m of an infected person for a cumulative total of 15 minutes or more over a 24-hour period (*6*, *7*). Symptoms (fever, headache, cough, indigestion) may appear as early as 5-6 days and as long as 14 days after possible exposure to the virus (*8*). Symptomatic and asymptomatic persons who have been in close contact with COVID-19 positive or suspected persons should be tested for COVID-19 to confirm transmission and existing infection (*9*). Asymptomatic individuals are considered silent transmitters, and those who have had no contact or are unaware that they have been in contact with a COVID-19 positive person are particularly important (*10*). Given the problem of silent transmission and the lack of conscious contacts, there is a need for screening tests that could indicate positive asymptomatic individuals who should be excluded from social interactions.

Currently, there are two types of diagnostic tests for detection of SARS-CoV-2 in the nasopharynx of infected individuals that indicate COVID-19. First and foremost are molecular tests that detect viral RNA sequences by nucleic acid amplification tests (NAAT), such as real-time reverse transcriptase-polymerase chain reaction (RT-PCR). This method is the gold standard for the detection of SARS-CoV-2, and results can later be confirmed by another NAAT assay or viral sequencing (*11*, *12*). Given the increasing number of COVID-19 cases and the urgent need to expand the capacity for COVID-19 testing during public health emergencies, there is a strong urge for cheaper, faster, and easier-to-use tests that could be used as screening or diagnostic tests for COVID-19 in broader populations. Therefore, another type of diagnostic test has recently been introduced, namely the lateral flow immunoassay (LFIA), which can detect the presence of specific viral antigens, *i.e.*, the spike (S) protein or the nucleocapsid (N) protein (*13*). These antigen tests aim to rapidly confirm the presence of SARS-CoV-2 in the nasopharyngeal swab of individuals with typical symptoms, as well as to carry out screening in a given population for epidemiological purposes, *i.e*., to determine how much the virus has spread in a community. In general, LFIAs provide the best information when a person is tested at the time of peak viral load and when exposure to a person with COVID-19 is known (*9*).

The first antigen test to receive Emergency Use Authorization (EUA) from the US Food and Drug Administration (FDA) on March 21, 2020 was the Cepheid Xpert Xpress SARS-CoV-2 test (*14*). Currently, more than 100 antigen tests are available for the detection of SARS-CoV-2 (*15*).

Lateral flow immunoassays have many advantages, such as relative ease of manufacture, ease of use, stability, and use of small sample volumes. One of the most appreciated advantages is that they are relatively inexpensive, making them available in many laboratories (*16*). On the other hand, there are also limitations in the performance of commonly used LFIAs, especially in terms of analytical sensitivity and test-to-test reproducibility. Test sensitivity may differ in terms of various antigen testing platforms. Laboratories should be aware of which platform is being used and the sensitivity of the test for the population being tested (*17*). For the interpretation of diagnostic tests, it is mandatory to have data on both their analytical and clinical sensitivity and specificity. Certainly, a prerequisite for diagnostic accuracy and reliability of diagnostic tests is their analytical accuracy and precision. The final clinical interpretation of antigen testing should include information on the presence of symptoms typical of COVID-19 and whether the patient has been in contact with a COVID-19 positive person.

In addition, COVID-19 patients could also be tested serologically for the detection of antibodies to SARS-CoV-2 (*11*, *18*). However, the clinical indications for serological testing and its analytical and clinical performance remain limited (*18*). Because seroconversion usually occurs two weeks after the onset of symptoms, serological testing should not be used as a diagnostic tool for acute COVID-19. Furthermore, the presence of SARS-CoV-2 antibodies does not indicate immunity to reinfection, but neutralization assays are needed (*18*).

This review aims to present the basic characteristics of LFIAs in the detection of SARS-CoV-2 in nasopharyngeal swabs during the COVID-19 pandemic from an analytical point of view, including the characteristics and requirements of the preanalytical, analytical, and post-analytical phases of the assay.

## Preanalytical phase

### Indication

**Figure 1:**
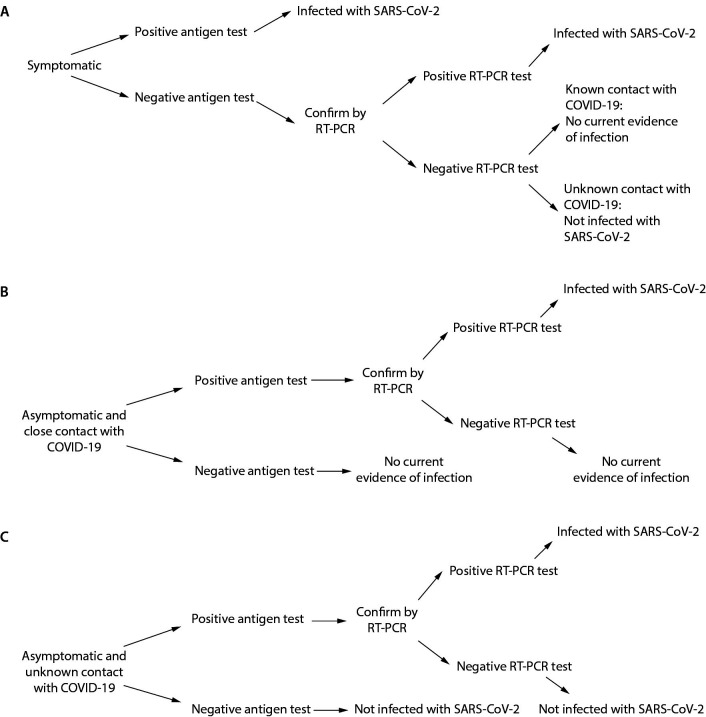
Antigen testing protocol and interpretation of results of infection with SARS-CoV-2: A) in a person with symptoms of disease; B) in an asymptomatic person who was in close contact with a COVID-19 positive person; C) in an asymptomatic person who was in unknown contact with a COVID-19 positive person. Adapted according to the reference (*9*). SARS-CoV-2 - severe acute respiratory syndrome coronavirus 2. COVID-19 - coronavirus disease 2019. RT-PCR - real-time reverse transcriptase-polymerase chain reaction.

The indication for LFIA is made depending on whether the patient tested is a person with severe symptoms of disease, an asymptomatic person who has been in close contact with the patient, or an asymptomatic person who is not aware that he or she has been in contact with an infected person (Figure 1) (*9*).

In addition, the result of the detection of SARS-CoV-2 in the nasopharyngeal swab depends on the clinical presentation, the anamnestic data of the individual and the time that has elapsed since contact with the infected person or patient. In most cases of patients with marked symptoms of disease, the result will be positive (*9*). Approximately seven days after the onset of symptoms, the amount of virus in the nose decreases, and after that period antigen tests may not confirm the presence of the virus (*9*, *19*). It is recommended to use antigen tests within five days of the onset of the disease (*20*). Despite the high specificity of antigen tests, false positive results may occur, especially when they are used in communities with low prevalence of infection.

### Nasopharyngeal swab sampling

Proper performance of nasopharyngeal sampling procedure requires competent and well-educated personnel (*e.g.,* nurses and technicians) who should be trained to always follow the same specimen collection steps. The patient’s head should be angled back approximately 70 degrees and the swab should be inserted perpendicular to the ear without lifting the swab so that it deeply contacts the nasopharyngeal wall. The swab should be rotated and rubbed against the nasopharyngeal wall (clockwise three to five times) and pulled out with circular motions to ensure that the absorbent tip of the swab is adequately saturated. Only if saturation is not satisfactory for any reason, such as difficult access due to blocked canals or septal deviation, should swabs be taken from both nostrils (*21*, *22*).

### Specimen handling and transport

Specimen handling is crucial in order to obtain a reliable result. Improper procedures can cause contamination of the sample. Specimens should be tested as soon as possible after collection (*9*). If previous is not possible, the sample is placed in a transport medium (with stirring for 15 seconds) and delivered promptly to the laboratory. Another important step in specimen handling after swabbing a potentially infected person is to handle the used materials as any other biohazardous materials in the laboratory (*23*).

Specimen handling, storage, and transport should be in accordance with the antigen test manufacturer’s instructions. Specimens are transported in special transport tubes that must be filled with 2-3 mL of liquid transport medium so that the absorbent tips are completely submerged. The most commonly used transport medium is viral transport medium (VTM). Viral transport medium contains the appropriate ingredients selected to create an isotonic solution, such as proteins required to protect the viral structure, antibiotics to control microbial contamination, and one or more buffers to control pH. Various VTMs are commercially available or can be prepared locally. The purpose of VTM is to provide appropriate temperature, pH, and nutrient conditions so that viability of the virus is maintained during storage or transport and drying of the sample is prevented. The applicability of VTM can be verified by culture isolation or directly by immunoassay (*24*). However, phosphate buffered saline (PBS), minimum essential media (MEM), or sterile saline also proved to be good options in the absence of standard VTM, as these solutions showed no loss of detection ability during antigen testing in seven-day period, whether refrigerated or frozen (*25*, *26*). Certain VTM formulations ensure inactivation of SARS-CoV-2 virus in only a few minutes (*27*). Only if the sample is tested immediately, it should not be placed in buffers or reagents (*22*, *28*). During transport, specimens should be wrapped with cold packs that maintain a temperature of 2-8 °C. Throughout transport, the packaging should maintain its integrity (*23*). Testing of specimens, that have been stored at 2-8 °C, should be performed within 72 hours of specimen collection (*29*).

## Analytical phase

### Principle of the analytical method

Lateral flow immunoassay is a qualitative point-of-care (POC) method that combines the principles of thin-layer chromatography and indirect immunochemical sandwich method with two antibodies, *i.e*., binding and detection antibodies (*30*). The stationary phase consists of a thin layer of adsorbent material (chromatographic paper or polymer) located on a flat, inert substrate. The binding antibody was immobilized on the chromatographic paper in a specific matrix, while the detection antibody labelled with colloidal gold was infiltrated into the sample pad. The mobile phase consists of a sample and buffer moving horizontally along the stationary phase, driven by capillary forces. When a sample is applied to the pad, the analyte forms an immune complex with the detection antibody labelled with colloidal gold. The sample moves laterally and forms a sandwich with the binding antibody. After a period of time, a coloured line forms at the site of the equivalence zone. The appearance of a coloured line indicates the presence of a targeted analyte in the sample. The appearance of a control line indicates that the analysis was performed properly (*31*). Tests from different manufacturers have different test performances and therefore different reading times. Most of the tests return results after 15 minutes. Test results should be read and interpreted at a specific time and not after the time manufacturer prescribed (*32*).

Although current LFIAs can detect either viral S or N antigens, the EUA and FDA have approved assays for the detection of N antigens (*13*). The antigen test result is expressed qualitatively, as positive or negative.

### Limit of detection

The limit of detection (LOD) of a method can be defined as the smallest amount of a substance that can be detected with reasonable certainty by a given analytical procedure (*33*). The LOD for N proteins ranges from 1.0 x 10^2^ TCID_50_/mL (TCID_50_ = fifty-percent-tissue-culture-infective-dose) to 4.5 x 10^5^ TCID_50_/mL. Lateral flow immunoassays cannot be used as the sole criterion for the detection of SARS-CoV-2 infection. Certainly, the positive result can only indicate the presence of viral N antigens in the sample, and the result can only be considered in the context of the patient’s clinical findings and other laboratory findings (*13*). False negative results may occur when the concentration of the target antigen in the clinical specimen is below the LOD value.

### Quality control

An internal control is already installed and included in the test device. It is represented by a coloured line in the control area. The appearance of the procedural control line indicates that sufficient flow has occurred and that the functional integrity of the test device has been maintained. If the procedural control line does not develop, the test result is considered invalid and retesting with a new device is recommended. However, internal controls do not evaluate proper sampling technique. Positive and negative external control swabs, usually included in the kit, should be run once with each new lot, shipment, and by each new user (*32*).

If the coloured line appears only within the control region, the result is negative. For the most antigen tests, the manufacturer’s instructions state that negative test results should be considered “presumptive”. This means that, in this case, the results are preliminary and the negative result should be verified by RT-PCR, especially if the person is showing any symptoms or has been in contact with a COVID-19 positive person. If the coloured line does not appear in the control area, the result should not be considered valid. If the control result is invalid, the antigen test must be repeated and such results must not be reported (*9*, *32*).

### Interferences

Interference can be defined as the effect of substances present in an analytical system that cause deviation of the measured value from the true value (*34*). Although interferences can affect the final results, especially in quantitative immunochemical methods, interferences can also manifest in qualitative immunoassays. Manufacturers of LFIAs for the detection of SARS-CoV-2 in nasopharyngeal swabs declared cross-reactivity of other viruses, bacteria, or yeasts (*Candida albicans*, 1 x 10^6^ TCID_50_/mL) and determined their cut-off values leading to possible interferences. A high dose hook effect may occur if the tested samples contain a concentration of SARS-CoV-2 virus higher than 1.6 x 10^5^ TCID_50_/mL (Table 1) (*35*).

**Table 1:** Possible interferences of viruses and bacteria in SARS-CoV-2 detection

**Potential reactants**	**Potential reactants**
**Viruses** Cut-off: 1 x 10^5^ TCID_50_/mL	**Bacteria** Cut-off: 1 x 10^6^ TCID_50_/mL
*Human coronavirus OC_43_*	*Bordetella pertusis*
*Human coronavirus 229E*	*Chlamydia pneumoniae*
*Human coronavirus NL_63_*	*Haemophilus influenzae*
*Rhinovirus*	*Legionella pneumophila*
*Adenovirus*	*Streptococcus pneumoniae*
*Human metapneumovirus*	*Streptococcus pyogenes* (Group A)
*Human parainfluenza virus 1*	*Mycobacterium tuberculosis*
*Human parainfluenza virus 2*	*Staphylococcus aureus*
*Human parainfluenza virus 3*	*Staphylococcus epidermidis*
*Human parainfluenza virus 4*	*Mycoplasma pneumoniae*
*Influenza A*	
*Influenza B*	
*Parainfluenza*	
*Enterovirus/Coxsackievirus B_4_*	
Adapted according to the reference (35). SARS-CoV-2 - severe acute respiratory syndrome coronavirus 2. TCID_50_ - fifty-percent-tissue-culture-infective-dose.	

Some substances that can be found in a patient’s nose may also cause cross-reactive reactions: phenylephrine, sodium chloride nasal gel, cromolyn, oxymetazoline, fluconazole, throat lozenge (benzocaine, menthol), fluticasone propionate, sore throat phenol spray (phenol), the antiviral drug Tamiflu (oseltamivir phosphate), mupirocin (antibiotic-nasal ointment), and tobramycin (antibacterial-systemic) (*35*).

Other limitations include the biotin interference aspect. Biotin levels of 2.5 µg/mL showed the interference and granted the false negative test results (*32*).

### Limitations

The test manufacturers warn of limitations of the LFIA which are important to consider when interpreting the results obtained. Some of these are: (i) a negative result occurs if the amount of antigen in a specimen is below the LOD of the test; (ii) unreliable results may appear when specimens are tested 1 hour after collection; (iii) the test detects both viable and non-viable SARS-CoV, and SARS-CoV-2; (iv) a positive result does not differentiate between SARS-CoV and SARS-CoV-2 (*35*).

## Postanalytical phase

### Clinical application and interpretation

A positive LFIA result in an individual who meets the clinical and epidemiological criteria for COVID-19 is considered sufficient to confirm the diagnosis of the disease. The obtained results, either positive or negative, must be entered into a separate information system platform that records only the results of LFIAs and not the results of molecular testing (*20*).

False positive results should be expected while screening large populations with a low prevalence of COVID-19 infection. A low prevalence is considered to exist if NAAT positivity in the past 14 days is less than 5% or if there are fewer than 20 new cases of COVID-19 per 100,000 persons within the past 14 days (*9*). In general, the lower the prevalence of infection in the community, the higher is the rate of false positive test results (*36*). False positive results can also be obtained due to cross-reactivity with the microorganisms containing N antigen, but also as a result of local application of drugs and preparations (*35*).

False negative results may be an outcome of improper sample collection if the amount of antigen in the sample is insufficient (*9*, *22*).

Comparison of rapid antigen tests with molecular methods for virus detection in nasopharyngeal swabs showed that antigen tests have lower sensitivity due to false negative results. This means that antigen tests should not be used as a frontline test for COVID-19 diagnosis (*37*).

In general, the sensitivity of antigen tests is lower than RT-PCR. Compared with RT-PCR, the first antigen tests that have received FDA/EUA approval have been shown to have a sensitivity of 80% to 97%, and the specificity is 100% (*38*). Recently published work showed that the accuracy of the Roche SARS-CoV-2 rapid antigen test compared with the molecular testing was 86.9%, while the sensitivity and specificity were 72.5% and 99.4%, respectively. In addition, the sensitivity was much higher (97-100%) in samples tested with cycle threshold (Ct) values of < 25 than in samples with Ct values between 30 and 37 (12-18%), proving that this test is reliable for the patients with a high viral load (*39*).

Antigen levels in specimens obtained later than 5-7 days after the first onset of symptoms may fall below the detection limit of the test. For this reason, the result may be falsely negative, whereas a more sensitive test, such as RT-PCR, may give a positive result (*9*). Since there is still no gold standard in antigen testing, the respective institutions report the analytical accuracy of the diagnostic tests they use by comparing them with validated tests. Further studies are needed to discern the overall performance of antigen tests (*40*).

It is recommended that the performance of antigen tests is validated in comparison with NAAT (*12*, *41*). Two respiratory swabs should be collected from each participant (within the first seven days of symptoms onset), one for molecular analysis and diagnosis and another for antigen testing. One swab may be sufficient if the buffer/transport medium is suitable for both methods. A minimum of 100 COVID-19 RT-PCR positives and 100 COVID-19 RT-PCR negatives should be included in the study. A sensitivity of 90% or greater and a minimum specificity of 97% are recommended (*41*). Antigen tests with acceptable performance can be included in a diagnostic algorithm to reduce the need for molecular testing (*12*).

Antigen tests showed high sensitivity and specificity in respiratory specimens from patients who were more symptomatic during the first week of infection with COVID-19 (*42*, *43*). The sensitivity of antigen tests is usually lower than RT-PCR, thus, negative antigen tests should be considered presumptive (*9*, *17*, *32*). Factors that increase the probability of confirming infection include the presence of symptoms in the person tested and recent exposure to a person diagnosed with COVID-19 (*17*). Tests on asymptomatic individuals have more false negative results than those on symptomatic individuals, which is only one limitation of antigen testing. Another limitation of antigen testing is that they deliver only a qualitative test, which does not provide insight into the viral concentration present in the sample. Furthermore, the prevalence of infection affects the predictive values of the antigen test. The positive and negative predictive values are significantly affected by disease prevalence. During peak activity, when disease prevalence is high, false negative test results are more likely. In contrast, during the period of low SARS-CoV-2 activity, while prevalence is moderate to low, there is a greater possibility of false positive test results (*32*).

In order to interpret the results correctly, several things should be considered, such as the performance characteristics of the antigen tests, the manufacturer’s instructions for use of the specific antigen test, and the prevalence of SARS-CoV-2 infection in the community. In addition, the clinical and epidemiological context of the person being tested must be recorded (*9*). Antigen tests provide the best results in cases with high viral loads, in pre-symptomatic and early symptomatic cases up to five days after the onset of the first symptoms (*41*). Since most approved antigen tests have high sensitivity and specificity, a positive result may be considered sufficient to confirm infection in symptomatic or contact-positive individuals, whereas RT-PCR should be performed in asymptomatic individuals and those without known viral exposure.

## Concluding remarks

During the COVID-19 pandemic, a central role in the health care system was given to the laboratory medicine. Lateral flow immunoassays are used in countries or areas where there is widespread population transmission, where the health care system may be overburdened, and where it is not feasible to test all or suspected cases by NAATs. Antigen testing has a major importance in the management of spreading COVID-19 infection in communities with high prevalence of SARS-CoV-2.

Despite anticipated limitations, LFIAs could play an important role in guiding patient management, public health decision making, and COVID-19 surveillance. There is a need to identify individuals with positive results as soon as possible to prevent further transmission of the virus through their self-isolation. Lateral flow immunoassays allow testing of a large number of individuals and obtaining a result in a short time and therefore have some advantage over RT-PCR. However, given the limitations of antigen tests, they cannot yet replace RT-PCR.

There is still little data in the literature on both the analytical and diagnostic validation of LFIA in the detection of SARS-CoV-2 in nasopharyngeal swabs. Furthermore, it could be assumed that without analytical accuracy of these rapid antigen tests, there is also no diagnostic accuracy and reliability. Future research is needed to further improve the analytical sensitivity and specificity of these rapid antigen tests so that the results can be compared to more expensive and complex molecular diagnostic tests.
